# *Baylisascaris procyonis* in the Metropolitan Atlanta Area

**DOI:** 10.3201/eid0912.020795

**Published:** 2003-12

**Authors:** Mark L. Eberhard, Eva K. Nace, Kimberly Y. Won, George A. Punkosdy, Henry S. Bishop, Stephanie P. Johnston

**Affiliations:** * Centers for Disease Control and Prevention, Atlanta, Georgia, USA

**Keywords:** *Baylisascaris*, raccoons, Georgia

## Abstract

*Baylisascaris procyonis*, the raccoon roundworm responsible for fatal larva migrans in humans, has long been thought to be absent from many regions in the southeastern United States. During spring 2002, 11 (22%) of 50 raccoons trapped in DeKalb County, Georgia, had *B. procyonis* infection. The increasing number of cases highlight this emerging zoonotic infection.

*Baylisascaris procyonis* was first described from specimens recovered from raccoons in Europe (*1*), although the first report of raccoon infections with the worm came from New York in 1933 (*2*). The first recognized human case was reported in 1984 in a 10-month-old child in Pennsylvania (*3*). Since then, at least 11 additional cases of severe or fatal *B. procyonis* encephalitis have been identified in Illinois, California, Michigan, Minnesota, New York, and Oregon (*4*).

The distribution of *B. procyonis* in the United States has been well recorded in some areas and poorly documented in other areas. The infection appears to be common in the Midwest, Northeast, and Middle Atlantic regions (*5*). Recently, *B. procyonis* has been found to be common in parts of California (*6*–*8*). However, *B. procyonis* historically has not been reported in the southeastern United States, except in mountainous areas. There is one anecdotal account of the infection in raccoons in central Georgia (*9*), and the literature cites an unpublished report of 1 out of 110 animals in north Georgia being infected (*10*), but no substantiated reports of the infection in Georgia have been found.

As part of ongoing studies that use animal dracunculiasis (*Dracunculus insignis*) as a model for the study of human dracunculiasis, raccoons were examined for preemergent female *D. insignis* worms. This study provided an opportunity to examine the animals for *B. procyonis*. We describe the occurrence of *B. procyonis* in the metropolitan Atlanta area (DeKalb County, Georgia).

## The Study

Raccoons (*Procyon lotor*) were trapped in residential areas by DeKalb County Animal Control personnel from March to June 2002, as part of the county’s nuisance animal abatement program. After animals were trapped, they were returned to the animal control facility, euthanized, and necropsied. The small intestine was removed, split open, and visually examined for *B. procyonis* worms. A stool sample was collected, placed in 10% formalin, returned to the Centers for Disease Control and Prevention (CDC) laboratories (where it was processed by using a standard formalin ethyl-acetate concentration procedure), and examined microscopically for *B. procyonis* eggs.

Of 50 raccoons examined during the spring of 2002, a total of 24 were female and 26 were male; all were adults. Eleven (22%) animals (5 female, 6 male) were found to be infected with *B. procyonis*. All 11 animals had detectable eggs in the feces. Worms were recovered from the small intestine in 8 of these. The number of worms recovered from individual animals ranged from 1 to 24, with a mean of 6.25 worms per animal, and all worms were preserved.

## Conclusions

Populations of raccoons harboring *B. procyonis* in and around major urban areas hold particular potential for zoonotic spread to humans. One reason is that raccoons adapt readily to human habitation and therefore tend to defecate in close proximity to homes (*11*), potentially putting large numbers of infective eggs in the immediate environment of children and others playing or working in yards, parks, playgrounds, and other similar environments. Heavily infected raccoons may shed millions of eggs daily, which is important because much human exposure to *Baylisascaris* is through the fecal-oral route and depends on the number of eggs in the environment (*4*,*11*,*12*).

To date, 12 human cases of infection with *B. procyonis* have been documented, and undoubtedly many more have been unrecognized. Four (30%) of these patients died, and the other patients were left with severe mental impairment. Most recognized cases, 10 of 12, have occurred in children 9 months to 6 years of age (*13*).

Until zoonotic diseases such as toxocariasis were actively sought with good serologic assays, their true occurrence was underestimated. The prevalence of *Baylisascaris* is undoubtedly greater than the number of reported cases would suggest, and the fact that the full clinical spectrum of illness for *Baylisascaris* infection has not been clearly elucidated, further lead to underrecognition of cases. Earlier case reports of diffuse unilateral subacute neuroretinitis or eosinophilic meningoencephalitis are compatible with *Baylisascaris* infection (*4*).

We cannot explain why *Baylisascaris* infection has turned up in the metropolitan Atlanta area at this time. Historically, the infection has been absent from this region of the southeastern United States, and surveys, including a number of our own unpublished observations in northern and southern areas of Georgia over the past 10 years, have never encountered this infection (*5*). During the preparation of this report, however, we received a call from a licensed wildlife rehabilitator who had received several young raccoons from Athens (Clarke County), Georgia, one of which had passed *Baylisascaris* worms. This person was well trained and very cognizant of *Baylisascaris* but had never seen the infection in any animals until this animal was received in June 2002. Geographic movement of other infectious diseases has been well documented; it is often linked to legal or illegal movement of natural host animals for a variety of purposes. We have no evidence of recent, large-scale movement of raccoons from enzootic areas into the metropolitan Atlanta area, but other explanations seem implausible. This may represent a natural migration of the parasite into new areas, but, again, no explanation of why this would be happening at this time is obvious.

This report highlights for clinicians and other public health officials, especially in the southeastern United States, the potential occurrence of *Baylisascaris* in an area previously thought to not be at risk and the need to be alert to the possibility of *Baylisascaris-*induced encephalitis, especially in young children. The outcome of this infection in humans is often fatal, but if the infection is recognized and treatment initiated early, larvae may be killed before they enter the central nervous system, thus mitigating the clinical disease or preventing death (*14*).
